# Development and Feasibility Assessment of a Rotational Orthosis for Walking with Arm Swing

**DOI:** 10.3389/fnins.2017.00032

**Published:** 2017-02-01

**Authors:** Juan Fang, Qing Xie, Guo-Yuan Yang, Le Xie

**Affiliations:** ^1^Jiangsu Key Laboratory of Advanced Food Manufacturing, Equipment and Technology, Jiangnan UniversityWuxi City, China; ^2^The Joint Lab of the Institute of Rehabilitation Centre and Chejing Robotics Technology (Shanghai) Co., Ltd., Med-X Research Institute, Shanghai Jiao Tong UniversityShanghai, China; ^3^Department of Rehabilitation Medicine of Ruijin Hospital, School of Medicine, Shanghai Jiao Tong UniversityShanghai, China; ^4^School of Material Science and Engineering, Shanghai Jiao Tong UniversityShanghai, China

**Keywords:** interlimb neural coupling, arm swing, normal gait, coordinated movement, rehabilitation robotics

## Abstract

Interlimb neural coupling might underlie human bipedal locomotion, which is reflected in the fact that people swing their arms synchronously with leg movement in normal gait. Therefore, arm swing should be included in gait training to provide coordinated interlimb performance. The present study aimed to develop a Rotational Orthosis for Walking with Arm Swing (ROWAS), and evaluate its feasibility from the perspectives of implementation, acceptability and responsiveness. We developed the mechanical structures of the ROWAS system in SolidWorks, and implemented the concept in a prototype. Normal gait data were used as the reference performance of the shoulder, hip, knee and ankle joints of the prototype. The ROWAS prototype was tested for function assessment and further evaluated using five able-bodied subjects for user feedback. The ROWAS prototype produced coordinated performance in the upper and lower limbs, with joint profiles similar to those occurring in normal gait. The subjects reported a stronger feeling of walking with arm swing than without. The ROWAS system was deemed feasible according to the formal assessment criteria.

## Introduction

People swing their arms synchronously with leg movement during walking due to interlimb neural linkage, in addition to mechanical factors. Although the arms have no direct function for propulsion (Barbeau et al., 1987), people normally swing their arms so as to improve gait stability (Behrman and Harkema, 2000; Bovonsunthonchai et al., 2012) and energy efficiency (Dietz, 2002; Collins et al., 2009). Apart from such behavioral relevance, many phenomena imply that arm swing during walking is a neural-coordinated motor output. Rhythmic muscle activity was observed in the constrained arms during walking overground (Eke-Okoro et al., 1997), which implies the existence of neural coupling between the upper and lower limbs. Furthermore, adding mass to one ankle induced adaptive changes in both arms, in addition to changes in EMG from the leg muscles (Donker et al., 2002). This resulted in a coordinated movement pattern similar to that seen in unloaded normal gait. Studies of walking on a split-belt treadmill with different speed ratios between the legs resulted in coordinated locomotion in the legs and arms (Dietz et al., 2001). Short accelerations or decelerations randomly applied to the right leg during treadmill walking produced EMG response in the bilateral arm muscles, in addition to that in the right leg (Dietz et al., 2001). Interlimb neural interaction thus appears to be an underlying neural mechanism of human bipedal locomotion.

The theory of interlimb neural coupling brings new requirements for gait rehabilitation robotics. Interlimb modulation is active during walking, but not in standing or sitting (Dietz et al., 2001; Zehr et al., 2012). The neural interaction between the upper and lower limbs is maintained in patients with injury to central nervous system (Visintin and Barbeau, 1994; Stephenson et al., 2010). The task-specific practice strategy suggests gait restoration robotic systems should provide locomotion–like movements to improve gait control and functional ability (Harkema, 2001). Judging from the implication of interlimb neural coupling and the fact of arm swing during walking, it was suggested that gait training after neurological injury should incorporate simultaneous upper limb and lower limb rhythmic exercise to take advantage of neural coupling (Ferris et al., 2006).

In spite of the existence of many types of rehabilitation robots, there is no system which activates both the upper and lower limbs in the same way as during walking. Over the last few decades many types of rehabilitation systems have emerged, including systems for gait restoration (Díaz et al., 2011) or for upper limb rehabilitation (Lum et al., 2005). Several lower-limb exoskeletons are commercially available to assist walking restoration, such as the Lokomat (Hidler et al., 2008) and the G-EO (Hesse et al., 2010) systems. They induce upright walking movement at variable speeds in the lower limbs. The arms often hold horizontal fixed bars to support the body. There are also several systems, such as the Armeo (Nef et al., 2006) and GENTLE/s (Loureiro et al., 2003), for those who have functional impairments in the upper limbs. The users often practice various arm movements in a sitting position. To the best of the authors' knowledge, there is no gait orthosis which incorporates arm swing.

Based on these limitations, a new rehabilitation system was to be developed in the present work. As the early initiation of gait rehabilitation is generally deemed important (Fang et al., 2011), the requirements of the proposed system included:

1. to allow the users to practice walking at the early post-injury stage;
2. to mimic the ground reaction forces on the foot which occur during walking;
3. to activate the upper limbs synchronously with the lower limb movement.

The aim of this work was to develop and evaluate the feasibility of a Rotational Orthosis for Walking with Arm Swing (ROWAS). The formal criteria for feasibility assessment were (Bowen et al., 2009): (i) implementation—was the system technically implementable? (ii) acceptability—was the system acceptable to the users? and (iii) responsiveness—was there a measurable movement that was close to the target joint trajectories?

## Methods

The starting point for this work was a gait analysis experiment to determine the target joint trajectories for the ROWAS system. After the ROWAS was developed and implemented as a prototype, a feasibility assessment was conducted using the formal feasibility criteria of implementation, acceptability and responsiveness.

### Gait analysis experiment

In order to provide reference data for the design of a system actuating both the upper and lower limbs, a gait experiment was performed using a Vicon motion analysis system (Oxford Metrics Ltd., Oxford, UK) in Ruijin Hospital, Shanghai Institute of Orthopaedics and Traumatology, China. Ethical approval was obtained from the Ethics Committee at the Med-X Research Institute, Shanghai Jiao Tong University, Shanghai, China. Twenty-four able-bodied subjects were recruited and they provided written informed consent prior to participation.

The experimental set-up consisted of a ten-camera Vicon MX13 motion capture system (Vicon Peak, Oxford, UK). Thirty-nine reflective markers of 14 mm diameter were affixed to specific anatomical landmarks (Plug-In Gait Marker Set, Vicon Peak, Oxford, UK) to record the segmental trajectories with a sampling frequency of 100 Hz (Kadaba et al., 1990). The details of this gait experiment can be found in our previous paper (Fang et al., 2016).

The angle trajectories of the shoulder, hip, knee and ankle joints within a gait cycle during overground walking were analyzed. The duration of one gait cycle was normalized to 100%, with heel strikes at 0 and 100%. The joint data were visually observed to remove outliers, and smoothed with the loess or rloess Matlab functions. The joint data from the overground walking experiment provided reference information of the joint profiles for the ROWAS system.

### Development of the ROWAS system

Based on the design requirements, the ROWAS system was implemented as a rotational bed linking frames for the upper and lower limbs. To achieve the required functional features, modules for a retractable wheel-set, a ground-simulation plate and a foot platform were further developed. A 3D CAD design software tool (SolidWorks, Version 2016, Dassault Systèmes SolidWorks Corporation, Massachusetts, the USA) was used to develop the structure of the ROWAS system.

#### Rotational bed frame

**Figure 1:**
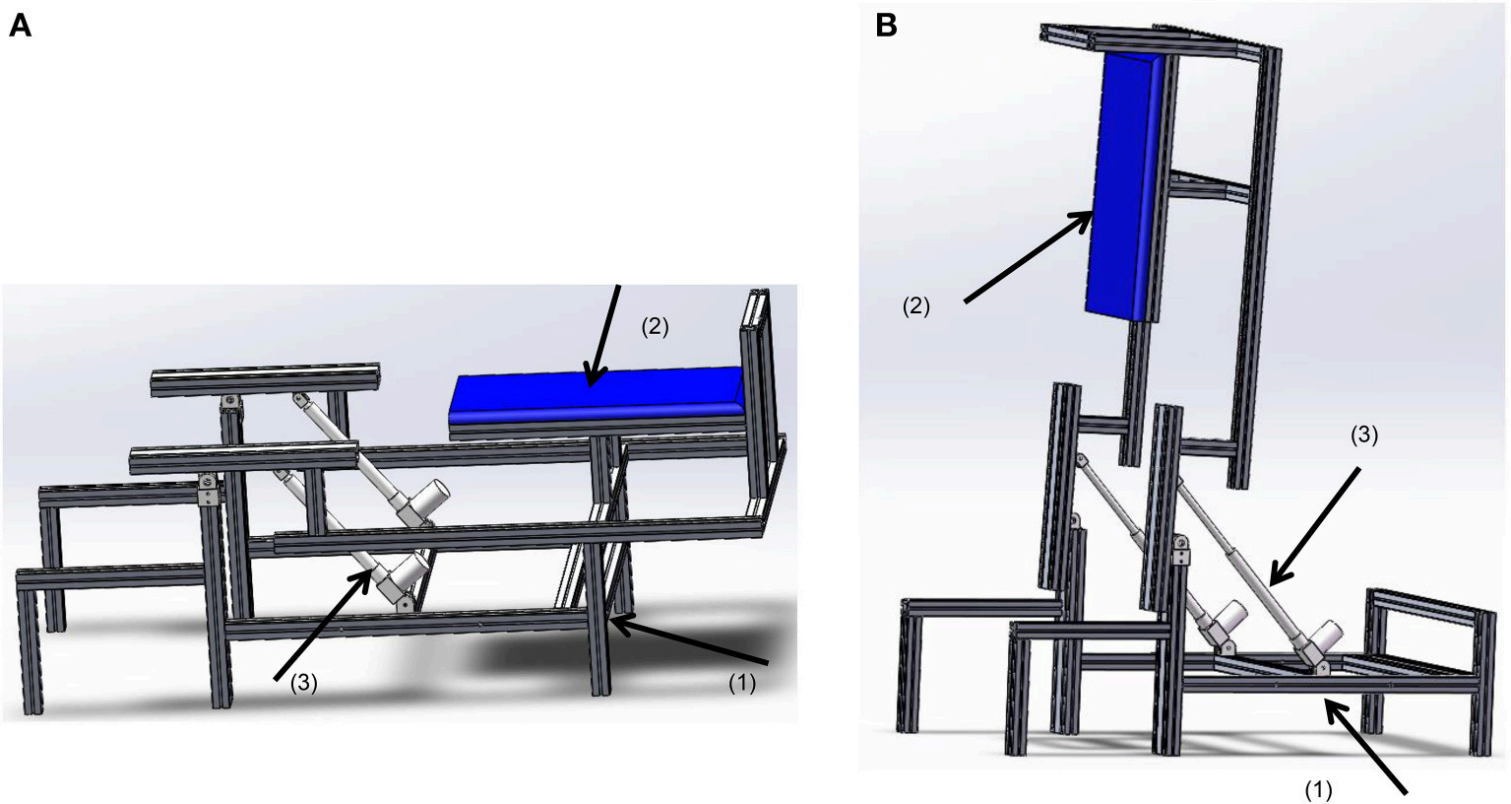
**The rotational bed**. (1) The bed base, (2) the bed plate, and (3) the linear actuator MD60. **(A)** In a lying position, **(B)** in an upright position.

In order to provide rehabilitation to users who are in an early post-injury phase, a rotational bed frame was developed, which allows practice in various positions. Depending on their physical situation, users can initiate walking training in a lying position (Figure 1A). Then as the rehabilitation progresses, the users can gradually be tilted up until in an upright position (Figure 1B).

**Figure 2:**
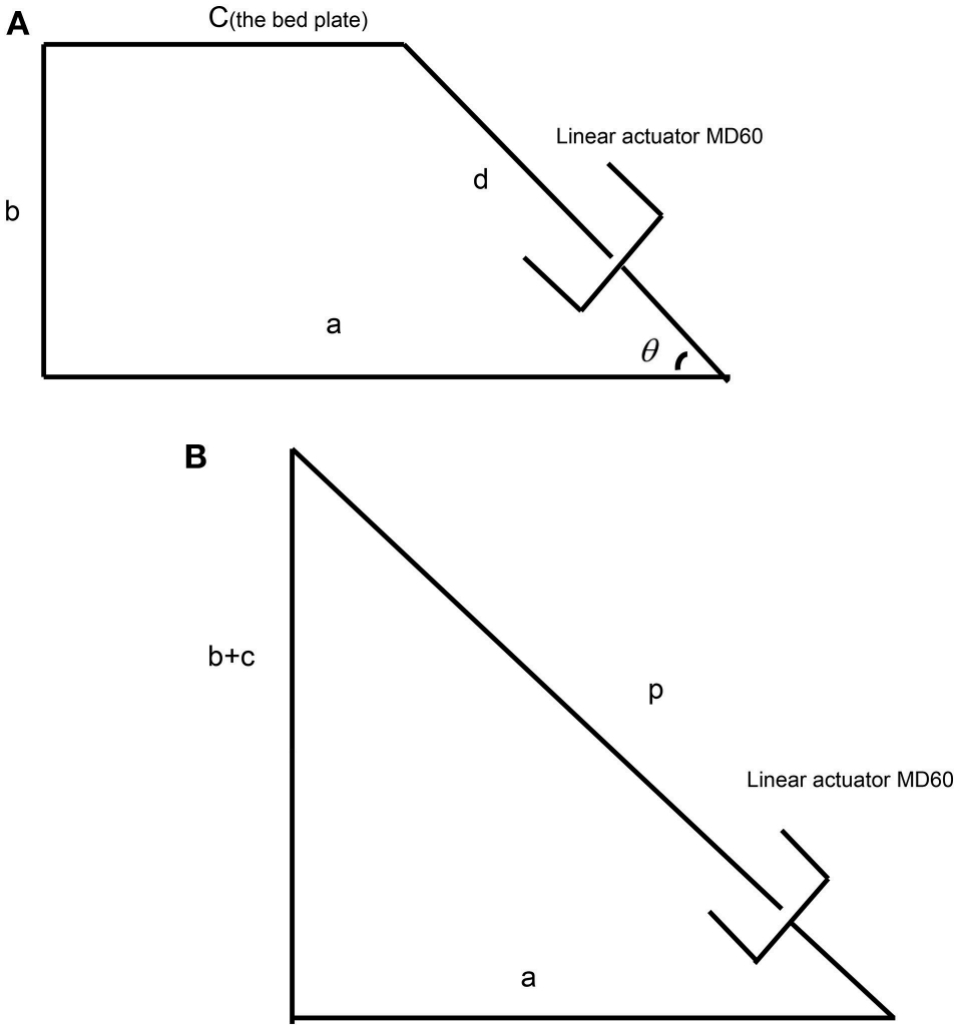
**The setup of the linear actuator for the simplified rotational bed. (A)** The actuator in the retracted state, **(B)** the actuator in the extended state.

We adopted a linear actuator MD60 from the Moteck Electric Corporation to rotate the bed up. As Figure 2 shows, the linear actuator allows the bed plate to be: (i) in a lying position when it is retracted to the shortest length *d*; (ii) in an upright position when it is extended to its longest length *p*. The following equations describe the device geometry:

(1) $$\begin{array}{r} d^{2}={\left(a-c\right)}^{2}+b^{2} \end{array}$$

(2) $$\begin{array}{r} p^{2}={\left(b+c\right)}^{2}+a^{2} \end{array}$$

(3) $$\begin{array}{r} \theta =\text{arctan}\frac{b}{a-c} \end{array}$$

We assumed the original height of the bed was 400 mm, i.e., *b* = 400. To secure an acceptable efficiency of the actuator, the angle of the actuator and the horizontal level θ should be larger than 45°. For the chosen linear actuator noted above, *stroke* = 300 mm; *d* = 530 mm; *p* = 830 mm; thus meeting these requirements. Using Equations (1) and (2), *a* = 560.2 mm and *c* = 212.5 mm. In this case, the angle between the linear actuator and the horizontal level θ was about 50°, which satisfies the above requirement.

#### Retractable wheel support

For ease of translation, a wheel-set was employed at the bottom of the bed frame. However, during gait training, the system should be supported by a stable base. Thus, a retractable wheel support was designed with the requirements: (i) to support the system when the system was to be moved; (ii) to allow the system to be supported by the bed leg when the system is in use for rehabilitation training.

**Figure 3:**
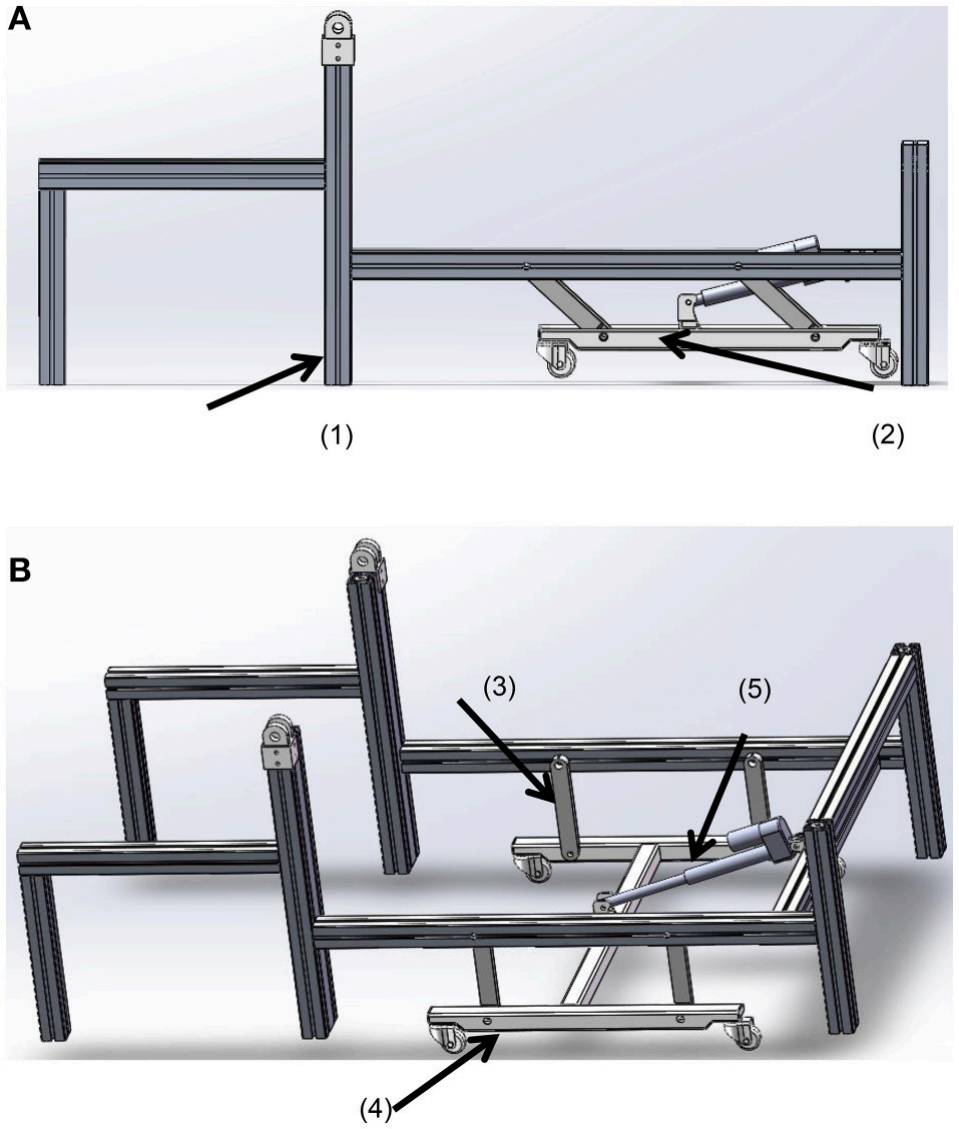
**The wheel-set under the bed. (A)** The retracted state; **(B)** the supporting state. (1) The bed leg, (2) the wheel-set, (3) the parallel bars, (4) the wheels, and (5) the linear actuator.

The wheel-set was attached by four parallel bars, which are lifted by a linear actuator. When the system is used for rehabilitation training, the wheel-set is retracted under the bed (Figure 3A) and the system is supported by the bed legs. When pushed by the linear actuator, the wheel-set was extended to the lowest position (Figure 3B), and the system is lifted up and supported by the wheels. The geometry for this linear actuator is similar to Equations (1)–(3). We also chose an MD21 from Moteck Electric Corporation, but with a stroke of 150 mm.

#### The ground-simulation plate and foot platform

**Figure 4:**
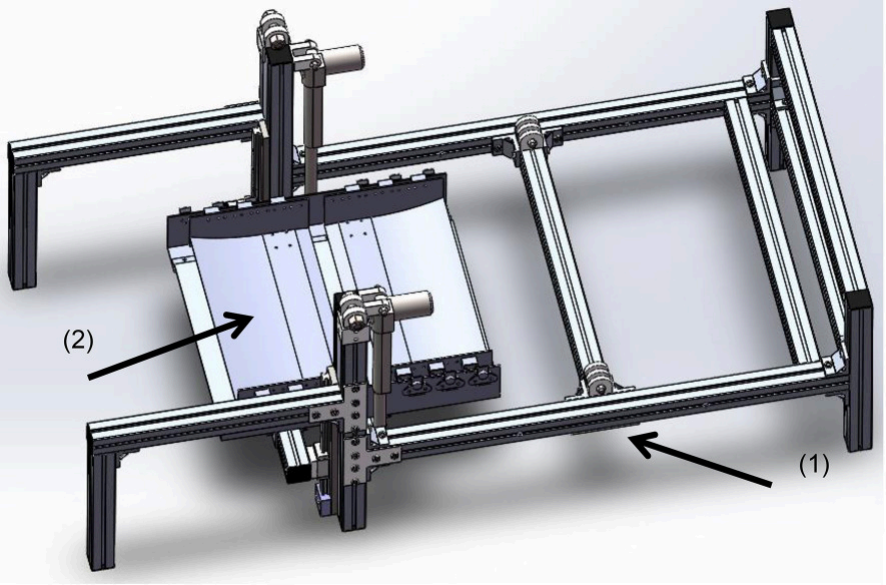
**The ground-simulation plate fixes on the bed base**. (1) The bed base, (2) the ground-simulation plate.

We designed a plate to support the leg during simulation of the stance phase. According to our systematic study on toe trajectories (Fang et al., 2016), it was found that the toe trajectory relative to the hip joint is curved. Therefore, the ground-simulation plate (Figure 4) was designed to have two ends taking a curved shape.

**Figure 5:**
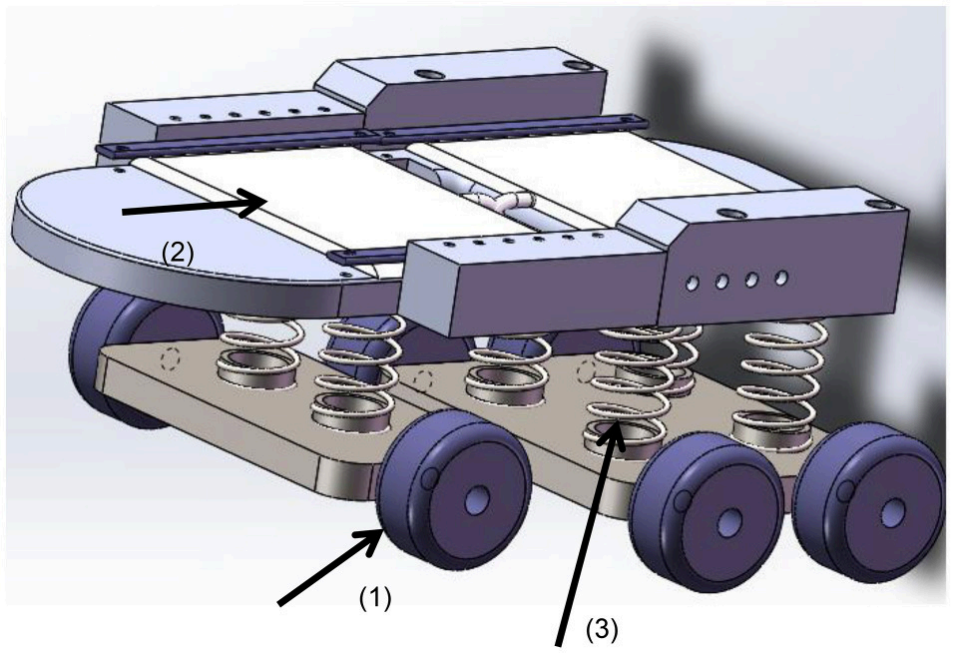
**The shoe platform**. (1) Wheels, (2) air bag, (3) springs.

In order to enable the subject to walk on the ground-simulation plate, we further designed a shoe platform (Figure 5) to support the foot and simulate the ground reaction force. With six wheels at the bottom, the shoe can roll on the ground-simulation plate. Two air bags were fixed on the shoe sole to simulate the events of heel-strike and toe-off. The springs between the foot base and the wheels were to provide space between the shoe platform and the ground-simulation plate.

#### Joint drives

**Figure 6:**
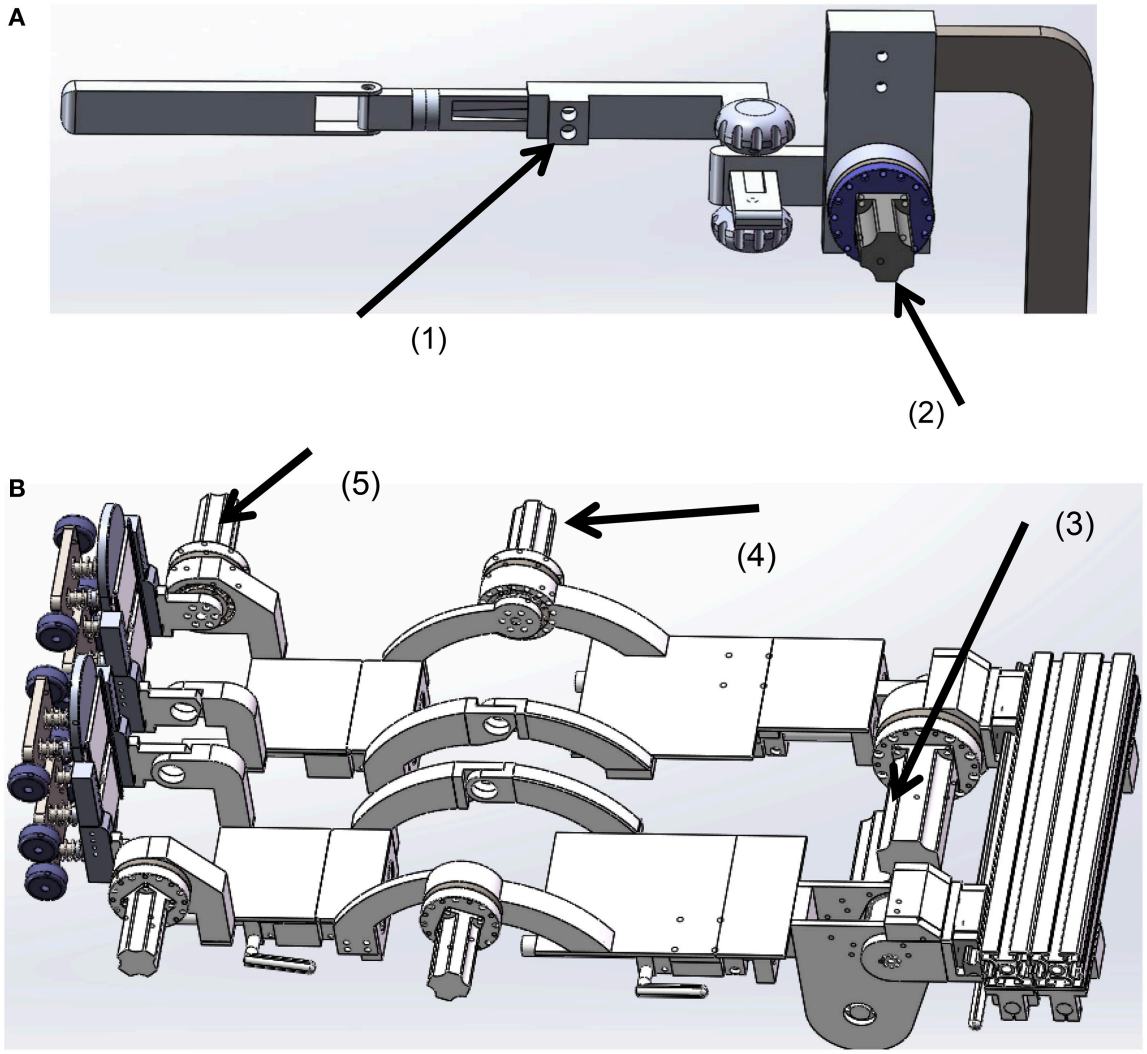
**The joint drives. (A)** The frame for the left upper limb, **(B)** the frame for the lower limbs. (1) The sliding nut with a clamp, (2) the shoulder drive, (3) the hip drive, (4) the knee drive, (5) the ankle drive.

For ease of control, we mounted the drives for the shoulder, knee, and ankle joints bilaterally at the sides (Figure 6). In order to get the upper limbs, especially the forearms, swing naturally as in normal walking, the hip joints require free space on each lateral side. Thus, we positioned the hip drives between the legs, as shown in Figure 6B. The drive for the right hip was mounted pointing to the joint center, while the drive for the left hip was mounted behind the left joint and the power was transmitted to the joint by a synchronous belt.

The rigs for the upper and lower limbs were designed adjustable in length to align the mechanical joints with the human joints. Size adjustment was implemented using a sliding nut that could be locked at any position on the frame by a clamp. The rigs for the lower limbs were covered with plates, which could support the leg during the transfer of the patient to the system.

#### The whole ROWAS system

**Figure 7:**
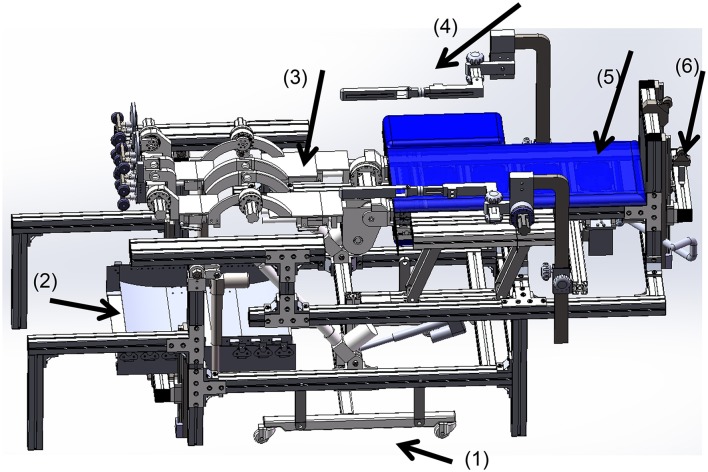
**Schematic presentation of the ROWAS system**. (1) The retractable wheel support (extended state), (2) the ground-simulation plate, (3) the rigs for the lower limbs, (4) the rigs for the upper limbs, (5) the rotational bed plate and (6) the body-weight support system.

With a wheel set support at the bottom, the system finally included two leg rigs and two upper limbs rigs which were fixed on a rotational bed (Figure 7). Secured by the body-weight support system, the users lie upon the bed. After their arms and legs are fixed to the upper and lower limb frames, the bed is tilted to a position where the users feel comfortable. Then the user starts walking training with arm swing. If the users can be tilted to an upright position, the shoe platforms roll on the ground simulation plate. When the system needs to be moved, the wheel-set can be lowered.

**Figure 8:**
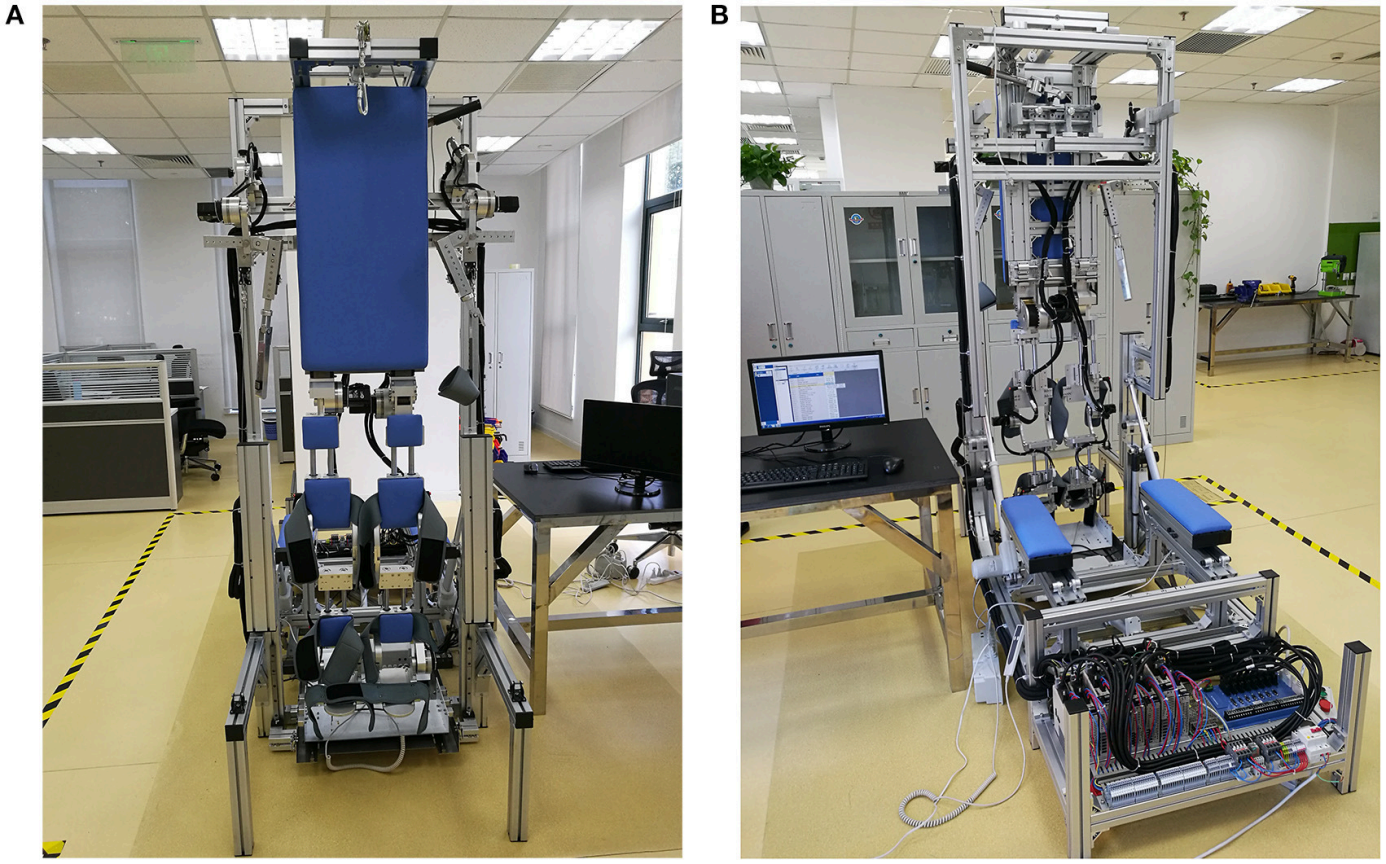
**The ROWAS prototype. (A)** The front view, **(B)** the back view.

The ROWAS prototype was manufactured as illustrated (Figure 8). Using strut profiles (Bosch Rexroth group), the bed frame was manufactured. The other structures were manufactured using aluminum alloy. The support frame and the joints of the ROWAS system had to be strong enough to transfer the necessary forces and torques to support a subject. To ensure this, the exoskeleton should be capable of developing the joint torques of a person of 135 kg walking at various speeds in various postures. For supine and upright walking, the maximal loads were derived from data presented in the literature (Fang et al., 2014a). The motors we selected were Yaskawa AC motors with harmonic gearboxes. The drive components for the two hip joints were a combination of a 400W AC motor (SGM7J-04AFC6S+SGD7S-2R8A00A) and a gearbox with ratio of 160 (LCSG-25-160), while the drive assemblies for the shoulder, knee and ankle joints were a combination of a 100W AC motor (SGM7J-01AFC6S+SGD7S-R90A00A) and a gearbox with ratio of 120 (LCSG-17-120).

An eight-axis Googol motion controller (GTS-800-PV-VB-PCI, GoogolTech Limited) was adopted to control the joint motors. The joint performance data from the overground experiment was the target movement for motors. To obtain satisfactory movement, the time course of each joint angle was fit using Fourier series functions. The fitted reference movements were sent to the corresponding motors via C++ software. Then the PID control algorithm imbedded in the Googol motion controller was employed to move the ROWAS prototype according to the target joint profiles.

### Experimental evaluation of the ROWAS prototype

Experimental evaluation had two subtests: a test on the prototype to assess only the system function, and tests on able-bodied subjects to collect user feedback on the prototype. As a preliminary trial on this novel training modality, we recruited five able-bodied subjects to evaluate the performance (Table 1). Ethical approval was obtained from the Ethics Committee at the Med-X Research Institute, Shanghai Jiao Tong University, Shanghai, China. Subjects provided written informed consent prior to participation.

**Table 1:** **Subject information**.

**Subject**	**Age (years)**	**Sex**	**Body mass (kg)**	**Height (m)**	**Thigh length (mm)**	**Shank length (mm)**
S1	32	F	55	1.60	390	350
S2	28	M	78	1.78	430	410
S3	28	M	60	1.70	410	370
S4	23	M	71	1.72	440	390
S5	29	M	66.7	1.72	430	380
Mean ± SD	28 ± 3.2	NA	66.1 ± 9.0	1.70 ± 0.065	420 ± 20	380 ± 22.4

#### Experimental procedure

During the experiment, the system produced walking at a cadence of 30 steps/min. In the preliminary test on the prototype only, the bed frame was rotated up at a speed of 1.5°/s to 75° relative to the ground. Then the system moved the frames for the upper and lower limbs. During the tests on the able-bodied subjects, we firstly collected their anthropometric data (Table 1). We then asked them to lie on the bed and secured them with the body-weight support system. After fixing their arms and lower limbs with Velcro straps, we rotated the bed to a position which was 75° relative to the ground. Finally we actuated the joint motors. The subjects tried to totally relax and follow the motion that the prototype produced. The test on each subject included two sessions: (i) the prototype actuated both the upper and lower limbs; (ii) it only moved the lower limbs, while the upper limb frames were left unactuated. Please see the Supplementary Video to see the performance of a representative subject during the test. The prototype produced walking for 45 s in each session. The joint performance was recorded via encoders. After the test, all the subjects were asked the questions below so as to collect their feedback:

1. Did you have any sense of insecurity during the process of bed rotation, walking with arm swing or walking without arm movement?
2. Did you feel any discomfort when using the system? If yes, please describe it.
3. Which type of motion did you feel was more like walking: walking with arm swing or walking without arm movement?
4. Do you have any suggestions for system improvement?

#### Data analysis

To compare the joint movements produced by the ROWAS prototype with those recorded during overground walking, three parameters were analyzed: difference of ROM (R_error_), phase shift (T_error_) and root-mean-square error (RMS_error_) for the angle performance (Fang et al., 2011, 2014b). The ROM of the hip joint from the overground walking experiment and the ROWAS test were R_OH_ and R_RH_, respectively, while the onsets of maximal hip extension in the overground walking experiment and the ROWAS test within one gait cycle T were T_OH_ and T_RH_. The angle amplitudes of the hip joint from the ROWAS and the overground walking were A_OH_ and A_RH_. The differences of the ROM, phase and RMS regarding the hip joint (R_errorH_, T_errorH_ and RMS_errorH_) between overground walking and the ROWAS test were obtained from Eqs (4-6). A similar procedure was adopted to compare the ROM difference, phase shift and RMS difference of the shoulder (R_errorS_, T_errorS_, RMS_errorS_), knee (R_errorK_, T_errorK_, RMS_errorK_) and ankle (R_errorA_, T_errorA_, RMS_errorA_) angle profiles from overground walking and the experiment. In order to obtain the phase shift of the shoulder and knee joints, the onsets of the maximal shoulder extension and knee flexion were analyzed. Thus,

(4) $$\begin{array}{r} R_{errorH}=\frac{R_{RH}-R_{OH}}{R_{OH}}\times 100‰ \end{array}$$

(5) $$\begin{array}{r} T_{errorH}=\frac{T_{RH}-T_{OH}}{T}\times 100‰ \end{array}$$

(6) $$\begin{array}{r} RMS_{errorH}=\frac{\sqrt{\frac{1}{N}\overset{N}{\underset{i=1}{\sum }}{\left(A_{RH}-A_{OH}\right)}^{2}}}{R_{OH}}\times 100‰ \end{array}$$

#### Criteria for feasibility assessment

The criteria employed for assessment of the feasibility of the ROWAS system were (Bowen et al., 2009): (i) implementation—was the system technically implementable? (ii) acceptability—was the system acceptable to the users? and (iii) responsiveness—was there a measurable movement that was close to the target joint trajectories? The ROWAS was considered to have satisfied responsiveness criteria if R_error_, T_error_, and RMS_error_ were less than 10%.

## Results

When the system was in a horizontal position, the dimension of the whole system was 2145 mm in length and 850 mm in width. The bed surface was 755 mm above the ground. It could be used by subjects with a height between 1.50 and 1.85 m. When the system was tilted up to be 90° relative to the ground (vertical), it was 2345 mm in height.

**Figure 9:**
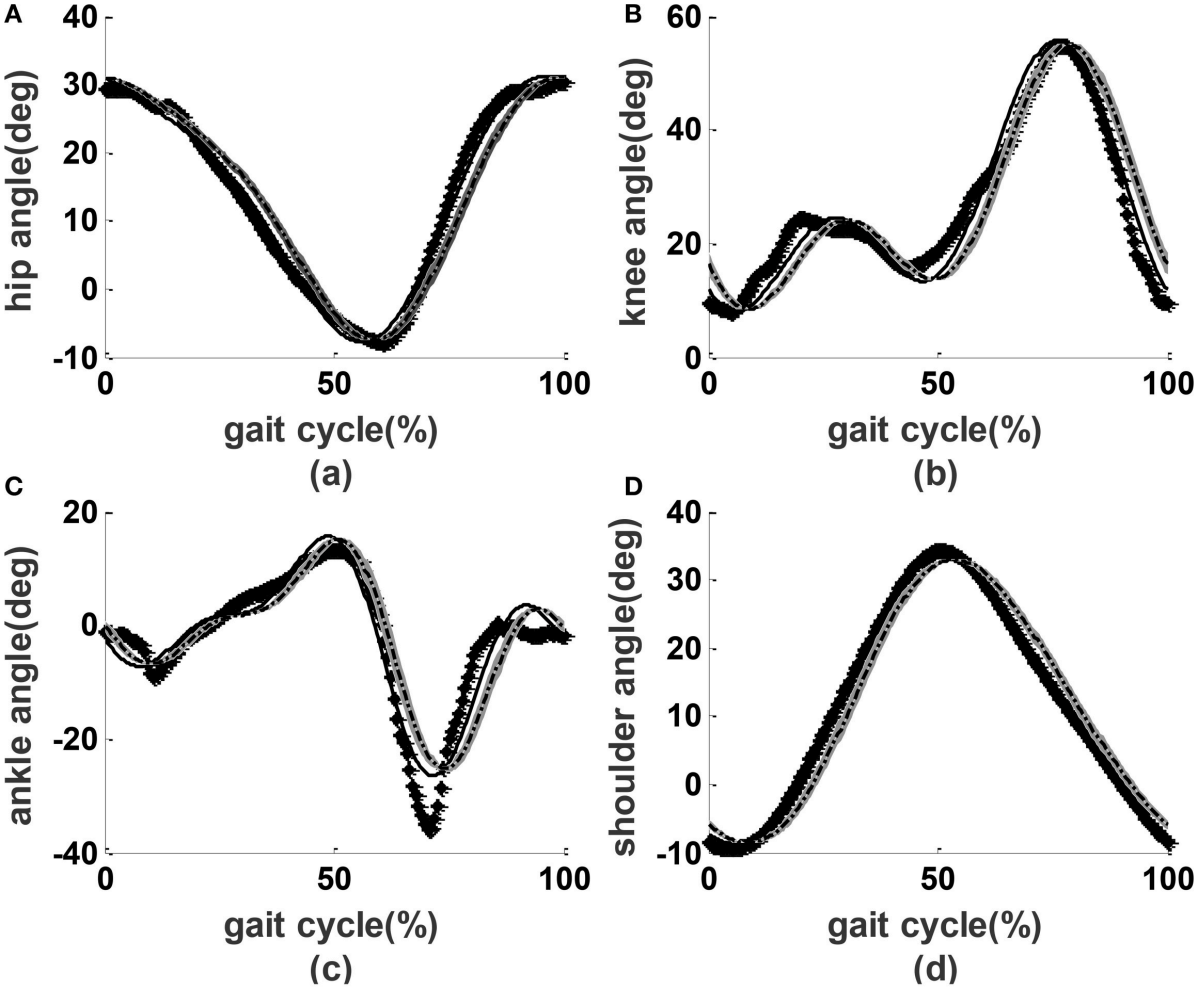
**Joint performance**. Lines with diamonds: measured experimental values; dashed lines: reference values; solid lines: the encoder readings from the prototype without users; dash-dot lines with shading: the mean ± SD performance from the prototype with users. **(A)** Hip angles, **(B)** knee angles, **(C)** ankle angles, **(D)** shoulder angles.

The target performance of the ROWAS system was taken from the gait experiment as described above. The joint angles of a representative subject with a height of 1.72 m were selected for illustration (the dotted lines in Figure 9). The fit data from the calculated Fourier functions served as the final reference values for the prototype (the dashed lines in Figure 9).

In the preliminary test on the prototype only, we applied a PID controller to actuate the joints. We observed that the parameters kp = 2 (proportional gain), ki = 0 (integral parameter) and kd = 0 (derivative parameter) provided acceptable results (the solid lines in Figure 9). During the test on able-bodied subjects, we applied the same PID parameters. The angle performance from the all subjects was close to that of preliminary tests on the prototype without users (dash-dot lines with shading in Figure 9). Compared to overground walking, the joint angle performance had a phase shift of about 3.5%, mean ROM errors of less than 8%, and RMS errors of less than 9% (Table 2).

**Table 2:** **The difference of the ROM error (%), the phase shift (%) and RMS error (%) between the ROWAS and overground data of all five subjects**.

**Joints**	**ROM deviation (*R_*error*_*)**	**Phase shift (*T_*error*_*)**	**RMS error (*RMS_*error*_*)**
Shoulder	5.23	−2.19	1.75
Hip	3.33	0.27	3.65
Knee	7.93	−0.28	6.32
Ankle	6.79	3.49	8.87

Feedback obtained from the subjects showed that S2, S4, and S5 had no sense of insecurity. S1 and S3 reported a sense of insecurity when tilted up, but not during walking sessions. All users reported a stronger sense of walking when the arms were actuated compared to when they were not. S2 and S4 said that the medial sides of both knee joints were somewhat uncomfortable. S1, S2, and S4 reported that the arm movement was not comfortable. S1 thought the step length should be smaller.

## Discussion

The aim of this work was to develop and evaluate the feasibility of the prototype ROWAS system. Feasibility assessment, as suggested by Bowen et al. (2009) and Saengsuwan et al. (2015), considered the formal criteria of implementation, acceptability and responsiveness.

The results demonstrated that the gait system with arm swing was technically implementable. Developed via computer modeling, ROWAS was implemented as a prototype. The main features were: (i) the system included a rotational bed which allowed the potential user to try various positions from supine to upright walking; (ii) the main support area was flat, such as the trunk support plate and the support plate for the lower limbs, which facilitates easy transfer of the users from their wheelchair to the system; (iii) the shoe platform produced ground reaction forces on the foot sole; and (vi) synchronous movement between the upper and lower limbs. Inclusion of arm swing could be simply implemented by adding the upper limb frames with drives. The challenge was that the arm swing function restricted the mounting options for the hip joint actuators. For ease of control, it was desirable for the drives to directly actuate the joints. However, arm swing required free space on both sides, especially around the hip joint area. Therefore, we put the hip drives between the legs. Furthermore, the ROWAS system was easy to move due to a retractable wheel support. The ROWAS prototype met the design requirements and is an innovative system.

The ROWAS system was tested regarding the acceptability criterion by the able-bodied subjects. Currently available gait orthoses normally have the arms fixed using a horizontal level bar for balance (Lum et al., 2005; Díaz et al., 2011). Recent research on interlimb neural coupling suggests that the inclusion of arm swing is helpful for gait restoration (Ferris et al., 2006). Therefore, the novel ROWAS system was developed with the inclusion of arm swing, and we were interested in testing whether this new training modality was accepted or not. Lying on the bed, and further secured by the body-weight support system, the subjects in the preliminary tests did not report any sense of insecurity during the walking session. After experiencing passive walking with and without arm swing in ROWAS, all the subjects reported a stronger sense of walking when arm movement was activated.

Regarding the responsiveness criterion, the test measurements proved that the prototype produced accurate responses close to the target profiles, i.e., a walking-like coordinated movement among the shoulder, hip, knee and ankle joints of both sides. Our study was motivated due to the absence of a gait system with arm swing. Although there are tricycles which produce movements in both the upper and lower limbs (Hunt et al., 2012), users are in a sitting position, with the limbs moving in a circular path. Thus, the movement profiles are quite different from those occurring during normal gait. The measured joint trajectories from our system demonstrated that a synchronized movement was produced among the upper and lower limbs, with a maximal phase shift of about 3.5%. Compared to overground normal walking, the mean ROM error of less than 8% and a maximal RMS error of 9% demonstrated that a natural walking style was generated in ROWAS. It was thus demonstrated that the ROWAS prototype was feasible regarding the responsiveness feasibility criterion.

Our study and the ROWAS prototype have some limitations. As a feasibility study, we only recruited five subjects. More subjects are desirable to obtain a more detailed evaluation of the prototype. Nevertheless, these subjects provided useful feedback on how to improve the ROWAS prototype for general application. S2 and S4 reported pressure around the knee joint. This was because the knee joint of the frame was not properly aligned with their knee joint. To address this limitation, we plan to add one more adjusting mechanism for the knee joint alignment. Three out of five subjects reported that the arm movement felt uncomfortable. This was because the shoulder joint was actuated in the sagittal plane only. We therefore plan to use a spherical joint to obtain more degrees of freedom. The ROWAS prototype had a fixed hip joint height, which was 1250 mm above the ground when the bed was in an upright position. People with different heights were aligned with the system via the hip joint. Therefore, shorter subjects (S1 and S3) had a sense of being lifted too high and had a sense of insecurity when tilted up. This insecurity resolved after the subjects got used to the height and it did not disturb their walking session. To solve this problem, the bed base could be improved to have an adjustable height. Another limitation was the fixed ROMs of the upper and lower limbs. Because of this, shorter users such as S1 thought the step length was too large. A variable walking style should be implemented.

Future work should prepare a modified ROWAS system for clinical tests on patients from target populations with neurological impairments. The height of the bed base should be reduced so that the patients can be easily transferred to the system from their wheel chair. To provide clinical testing, we need to design a better body-weight support system which secures them, but without discomfort in the bottom area. Finally, the control algorithms need to be extended to allow compliant movement, and also to allow potential subjects, especially hemiplegic patients, to use their healthy side to control the impaired side. EMG responses should be investigated when patients use the system, as this will allow further investigation of interlimb neural coupling.

## Conclusions

In conclusion, we designed, manufactured and tested a ROWAS. During testing with five able-bodied subjects, the system could produce coordinated joint performance in the upper and lower limbs which was similar to normal overground gait. This system was deemed feasible, as far as the formal implementation, acceptability and responsiveness criteria were concerned.

## Author contributions

JF mainly developed this project, designed the system, manufactured and tested the system, analyzed the data and wrote the paper; QX helped develop the concept of this project and modified the manuscript; GY developed this project, supervised the experiments and analyzed the data and checked the paper; LX developed this project, helped to develop the design concepts, analyzed the data and checked the paper.

### Conflict of interest statement

The authors declare that the research was conducted in the absence of any commercial or financial relationships that could be construed as a potential conflict of interest.
